# The Health Promoting Bioactivities of *Lactuca sativa* can be Enhanced by Genetic Modulation of Plant Secondary Metabolites

**DOI:** 10.3390/metabo9050097

**Published:** 2019-05-12

**Authors:** Hammad Ismail, Anna L. Gillespie, Danielle Calderwood, Haroon Iqbal, Colene Gallagher, Olivier P. Chevallier, Christopher T. Elliott, Xiaobei Pan, Bushra Mirza, Brian D. Green

**Affiliations:** 1 Department of Biochemistry, Quaid-I-Azam University, Islamabad 45320, Pakistan; hammadismail5@gmail.com (H.I.); bushramirza@qau.edu.pk (B.M.); 2 Institute for Global Food Security, School of Biological Sciences, Queen’s University Belfast, Biological Sciences Building, Chlorine Gardens, Belfast BT9 5DL, UK; agillespie14@qub.ac.uk (A.L.G.); dcalderwood01@qub.ac.uk (D.C.); hiqbal02@qub.ac.uk (H.I.); cgallagher58@qub.ac.uk (C.G.); o.chevallier@qub.ac.uk (O.P.C.); Chris.Elliott@qub.ac.uk (C.T.E.); xpan1020@gmail.com (X.P.); 3 Core Technology Unit for Mass Spectrometry, Faculty of Medicine, Health and Life Sciences, Queen’s University Belfast, Belfast BT9 5DL, UK

**Keywords:** Lettuce, plants, secondary metabolites, metabolomics, diabetes

## Abstract

Plant secondary metabolites are protective dietary constituents and *rol* genes evidently increase the synthesis of these versatile phytochemicals. This study subjected a globally important vegetable, lettuce (*Lactuca sativa*) to a combination of untargeted metabolomics (LC-QTof-MS) and in vitro bioactivity assays. Specifically, we examined the differences between untransformed cultured lettuce (UnT), lettuce transformed with either *rolABC* (RA) or *rolC* (RC) and commercially grown (COM) lettuce. Of the 5333 metabolite features aligned, deconvoluted and quantified 3637, 1792 and 3737 significantly differed in RA, RC and COM, respectively, compared with UnT. In all cases the number of downregulated metabolites exceeded the number increased. In vitro bioactivity assays showed that RA and RC (but not COM) significantly improved the ability of *L. sativa* to inhibit α-glucosidase, inhibit dipeptidyl peptidase-4 (DPP-4) and stimulate GLP-1 secretion. We putatively identified 76 lettuce metabolites (sesquiterpene lactones, non-phenolic and phenolic compounds) some of which were altered by several thousand percent in RA and RC. Ferulic acid levels increased 3033–9777%, aminooxononanoic acid increased 1141–1803% and 2,3,5,4′tetrahydroxystilbene-2-O-β-d-glucoside increased 40,272–48,008%. Compound activities were confirmed using commercially obtained standards. In conclusion, *rol* gene transformation significantly alters the metabolome of *L.sativa* and enhances its antidiabetic properties. There is considerable potential to exploit *rol* genes to modulate secondary metabolite production for the development of novel functional foods. This investigation serves as a new paradigm whereby genetic manipulation, metabolomic analysis and bioactivity techniques can be combined to enable the discovery of novel natural bioactives and determine the functional significance of plant metabolites.

## 1. Introduction

The *rol* genes (*rolA, rolB, rolC* and *rolD*) are plant oncogenes which induce root formation in transformed plant cells [1,2]. Extensive efforts have been made to understand how these genes affect plant growth, development and hormone metabolism [3]. However, a novel and recent discovery is the apparent ability of the *rol* genes to increase a plant’s production of secondary metabolites [3]. Transforming plants or plant cell cultures with *rolC* stimulates the production of alkaloids, ginsenosides and anthraquinones [4,5,6,7]. The combined effects of *rolA, B* and *C* genes has also been examined, with increases in secondary metabolites observed in the hairy roots of most transformed plant species [8]. Plant secondary metabolites are structurally diverse phytochemicals deemed to be ‘non-essential’ (normally defined as not involved in photosynthesis, respiration, growth or development). A highly topical area of human nutrition research is the investigation of these highly versatile natural products as protective dietary constituents. There is increasing evidence that they have beneficial effects within the context of diabetes, cardiovascular disease and cancer, also that long-term modest intake could reduce the incidence of disease [9]. Furthermore, the absorption of many phytochemicals such as phenolic compounds (e.g., hydroxycinnamic acids) and flavonoids (e.g., chicoric acid) has been demonstrated in humans [10,11]. The development of metabolomics as a scientific discipline (along with major advances in analytical instrumentation) now make it possible perform detailed assessments of how plant metabolites are affected by genetic manipulation, agronomical strategies and environmental factors [12].

Investigations have shown that polyphenolic compounds are able to regulate postprandial glycaemia and inhibit the development of glucose intolerance by stimulating secretion of GLP-1 [13,14]. The present study examined a globally important leafy vegetable, lettuce (*Lactuca sativa*), as a model for studying the effect of *rol* genes on plant metabolites. Lettuce contains valuable nutrients such as polyphenols, sterols, vitamins, minerals and dietary fibre. Many of lettuce’s metabolites and phytochemicals are associated with antioxidant and health-promoting properties and lettuce has even been used in folk medicine [15,16,17]. For instance, sesquiterpene lactones from lettuce appear to have analgesic, anti-inflammatory and sedative properties [18,19]. Consumption of lettuce has potential for curbing human metabolic disorders since it improves cholesterol metabolism and antioxidant status [17], however, it has not yet been determined whether lettuce possesses therapeutic potential for type 2 diabetes mellitus. Nonetheless, lettuce would appear to be a useful model system for investigating the functional effects of plant secondary metabolites.

We used untargeted high resolution mass spectrometry (LC-QTof-MS) metabolomic analysis to broadly assess the impact of *rol ABC* (RA) and *rol C* (RC) transformation on the primary and secondary metabolites of *L.sativa* (cv. Grand Rapids). We also examined the functional benefits of *rol* gene transformation by assessing changes in in vitro bioactivity. The specific bioactivities tested were inhibition of α-glucosidase, inhibition of dipeptidyl peptidase-4 (DPP-4), and stimulation of glucagon-like peptide-1 (GLP-1) secretion (an important human intestinal hormone). These 3 anti-diabetic targets are the basis of pharmacological treatments for type 2 diabetes mellitus and it is well established that they can be modulated by natural products, nutrients and phytochemicals [14,20,21,22,23,24,25,26].

## 2. Results

### 2.1. Agrobacterium Rhizogenes rol Genes Enhance the Antidiabetic Bioactivity of Lactuca sativa

**Figure 1:**
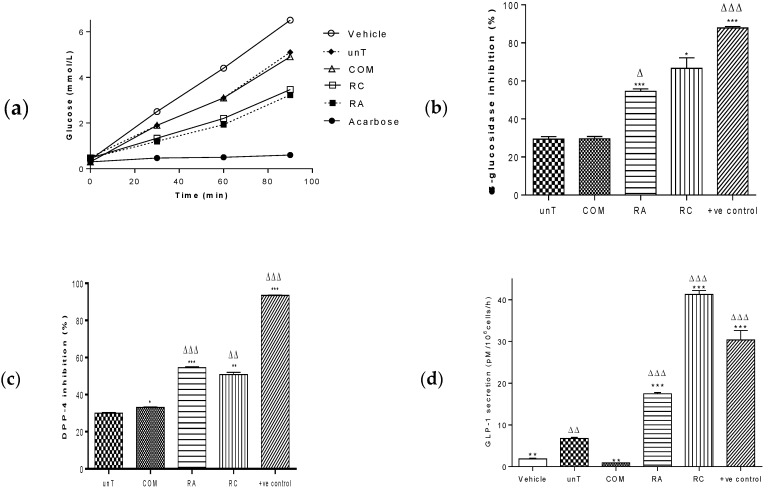
Bioactivity of *rol* gene transformed *L. sativa*. α-glucosidase inhibition by *L. sativa*. (**a**) Liberation of glucose from maltose over time and (**b**) percentage α-glucosidase inhibitory for each L. sativa extract (0.25–1.0 mg/mL; *n* = 3). Positive control was acarbose (1 mg/mL) (**c**) DPP-4 inhibition for each L. sativa extract (0.25–1.0 mg/mL; *n* = 3). Positive control was berberine (1 mg/mL) (**d**) GLP-1 secretory responses of each L. sativa extract (1.0 mg/mL; *n* = 4). Positive control was α-lactalbumin (10 mg/mL). All values are mean ± SEM. All statistical comparisons were One Way ANOVA. * *p* < 0.05, ** *p* < 0.01 and *** *p* < 0.001 compared with UnT ∆ *p* < 0.05, ∆∆ *p* < 0.01 and ∆∆∆ *p* < 0.001 compared with COM. unT = untransformed cultured; COM = commercially obtained; RA = *rol ABC* transformed and RC = *rol C* transformed. Inhibitory activities (α-glucosidase and DPP-4) were calculated as a percentage of the vehicle control.

Untransformed *L. sativa* possessed inhibitory activity against both α-glucosidase and DPP-4 enzymes, as well as stimulating the secretion of GLP-1 in an enteroendocrine cell line. Both *rol ABC* (Figure 1a,b; RA; 55%; *p* < 0.001) and *rol C* (RC; 67%; *p* < 0.01) transformed *L. sativa* possessed higher levels of α-glucosidase activity than unT (29%). Similarly, RA (55%) and RC (51%) possessed greater DPP-4 inhibitory activity than unT (30%) *L. sativa* (Figure 1c). The GLP-1 secretory activity of *rol* gene transforms was particularly impressive (Figure 1d). Secretory activity of RA was 260% (*p* < 0.001) greater than unT and RC was 616% (*p* < 0.001) greater. Commercially obtained (COM) *L. sativa* had broadly similar levels of α-glucosidase inhibitory activity as UnT (30 vs 29%), modestly increased levels of DPP-4 inhibitory activity (33% vs 30%; *P* < 0.05) as UnT. Contrastingly, COM had 86% (*p* < 0.001) lower GLP-1 secretory activity than UnT, which was lower than even the vehicle control.

### 2.2. Agrobacterium rhizogenes rol Genes Induce Widespread Changes in the L. sativa Metabolome

**Figure 2:**
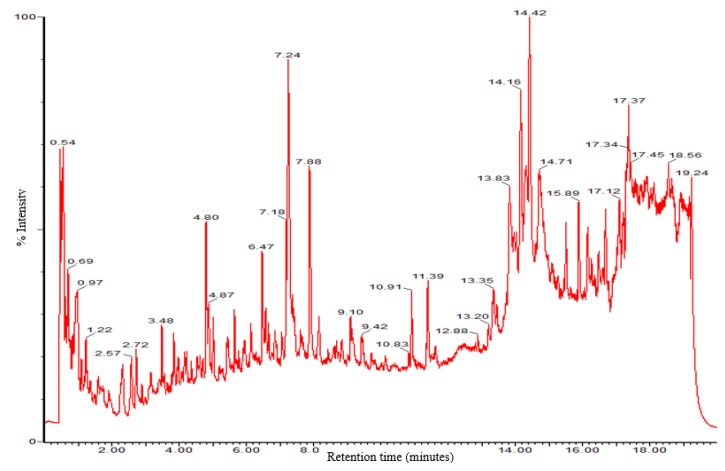
Chromatographic separation of *L. sativa* extracts. Reversed-phase UPLC ESI− chromatogram of the methanol extract of *L. sativa*. Each chromatogram contained a total ion count (TIC) of 5333 aligned and deconvoluted features.

Untargeted LC-QTof-MS analysis investigated the metabolomic differences in transformed (RA & RC), untransformed (UnT) and commercially (COM) obtained *L. sativa* (cv Grand Rapids). Both ESI− and ESI+ acquisition modes were assessed during method development. However, ESI− consistently produced much greater numbers of ions of stronger intensity than ESI+ and therefore ESI− was used. A typical base peak chromatogram (BPC) for *Lactuca sativa* is shown in Figure 2.

**Figure 3:**
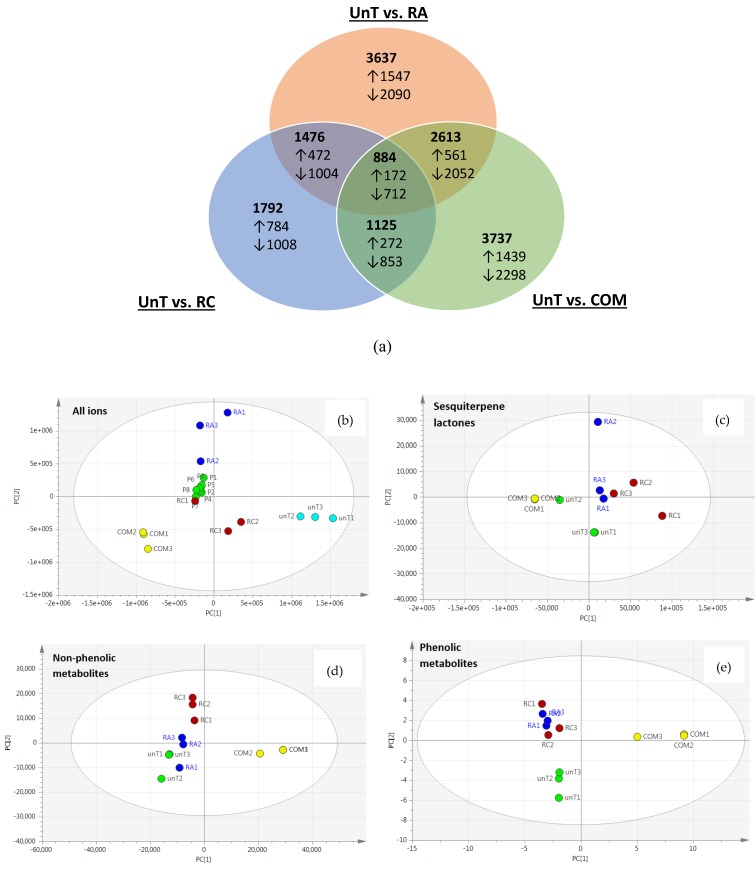
Analysis of LC-MS metabolomic data. (**a**) Shows the number of the 5333 ions/metabolites measured which were significantly different (*p* < 0.05) in group comparisons. All *L. sativa* groups were compared simultaneously (Principal Component Analysis (PCA)) to build a model encompassing (**b**) all 5333 detected metabolites, (**c**) sesquiterpene lactone metabolites (12), (**d**) non-phenolic metabolites (24) and (**e**) phenolic metabolites (40). Each *L. sativa* group is indicated with: unT: untransformed tissue culture plants (light blue); COM: commercially purchased plants (yellow); RA: *rol ABC* transformed plants (dark blue); and RC: *rol C* transformed plants (red). Pooled (P; green) samples included quality control purposes are shown in (**b**).

Of the 5333 aligned and deconvoluted features that there were many significant differences which overlapped UnT, RA, RC and COM (Venn diagram; Figure 3a). The number of metabolites which significantly differed between UnT vs. RA and UnT vs. COM (3637 and 3737, respectively) was similar, and of these there were 2613 differences which were shared. In contrast, UnT vs. RC had much fewer significant differences (1792), but a large proportion of these metabolites overlapped with UnT vs. RA (1476) and UnT vs. COM (1125). Surprisingly, in all cases the number of downregulated metabolites exceeded the number which were increased. Unsupervised Principal component analysis (PCA) of all 5333 ions revealed distinct groupings with UnT, COM, RC and RA each clustered into distinct regions of the PCA plot indicating that that metabolomic profile differed substantially from one another (Figure 3b). The first two principal components accounted for over 70% of the variation in the data, with principal component 1 (PC1) accounting for the majority of the variation (50 %) and PC 2 (32 %). It was also clear that in each individual genetic transformation event increased metabolomic variability, since RA1, RA2, RA3 and RC1, RC2, RC3 were spread more widely than biological replicates of either UnT or COM.

Using the exact mass and fragmentation data in combination with online databases and published literature [12] we manually identified 76 known metabolites (Properties detailed in Tables S2–S4). These 76 putative identifications all had theoretical and observed M-H^−^ masses differing by less than 0.001 Da, had a mass error no more than 20 ppm, and had been previously been reported as occurring in *L.sativa*. The 76 were classified as either sesquiterpene lactones (12), phenolic compounds (40) or non-phenolic compounds (24). Using PCA we assessed how each of these classes were influence by *rol* gene transformation (Figure 3b–d). This initial assessment indicated that *rol* gene transformation clearly induced changes in each class of compounds. However, non-phenolic (Figure 3c) and phenolic compounds (Figure 3d) in particular appeared to differ more in COM (i.e., separated across PC1 rather than PC2). This led us to compare how the levels of each individual metabolite differed.

### 2.3. Sesquiterpene Lactones and Related Derivatives/Conjugates

Of the 12 sesquiterpene lactones identified 10 significantly differed in COM, 7 differed in RA and 9 differed in RC (Table 1). Levels of lactucin were unaffected by *rol* genes but COM had significantly higher levels (667%). Despite this there was evidence that RA and RC had a greater propensity to affect the levels of some sulfated lactucin metabolites. For example, 15-deoxylactucin-8-sulfate-Val was increased by 1103% and 1028% by RA and RC, respectively, but was unchanged in COM. Similarly, 5-deoxylactucin-8-sulfate-Gln was increased by 535% and 511% by RA and RC, respectively, but was 895 lower in COM. The amount of 1-β-(4-hydroxyphenylacetyl)-15-O-β-d-glucopyranosyl-5α-6βH-eudesma-3-en-12-6α-olide differed across all 3 groups with higher levels in RA (327%), decreased levels in COM (100%) and no significant change in RC. On the other hand, levels of some metabolites were consistently higher across groups, for example 11β,13-dihydrolactucopicrin was increased by 132%, 257%, and 127% in RA, RC and COM, respectively (Table 1).

### 2.4. Non-Phenolic Metabolites

Of the 24 non-phenolic metabolites identified 22 significantly differed in COM, 18 differed in RA and 15 differed in RC (Table 2). The majority of these were either amino acids, dipeptides, alkaloids or organic acids, but we also encountered the vitamin pantothenic acid (B5). Many of these would be considered primary (not secondary) metabolites and in nearly all cases these were more abundant in COM than the cultured plants. This for example included amino acids (Leu/Ile: 794%, Trp: 494% and Phe: 2968), organic acids (isopropylmalic acid: 537% and citric acid: 1137%), alkaloids (1,2,3,4-Tetrahydro-beta-carboline-3-carboxylic: 1698% and 1,2,3,4-Tetrahydro-b-carboline-1,3-dicarboxylic acid: 1567%), the nucleoside adenosine (224%) and the dipeptide Gly-Leu (289%). Sugar conjugates of amino acids (Trp-hexose, Tyr-hexose, Phe-hexose and glycine-leu/Ile-hexose) were also much more abundant in COM (1500%–2900%). The few exceptions to this were amino oxononanoic acid which had higher levels in RA (1803%) and RC (1141%) than COM (188%), but also the purine nucleoside guanosine which had higher levels in RA (200%) and RC (255%) than COM (113%).

### 2.5. Phenolic Metabolites

Of the 40 phenolic metabolites identified 31 significantly differed in COM, 26 differed in RA and 22 differed in RC (Table 3). Among these identified compounds 3-Methoxy-4-hydroxyphenyl-1-O-β-D-glucoside was higher in RA (115%) and RC plants (153%) than COM (28%). There were many examples of phenolic compounds which were higher in COM including dihydroxybenzoic acid (70%), dihydroxybenzoic acid (223%), caffeic acid (326%), p-coumaroyl glucoside (880%), caftaric acid (2290%), caffeoyltartaric-p-coumaroyl acid (822%) and p-coumaroylquinic acid (3978). Transformation has enhanced the ferulic acid methyl ester about 5616% and 9777% in RA and RC plants respectively. This increase is the highest among the above stated group representation the strong positive effect of *rol* gene in in the production of secondary metabolites. Another compound di-4-hydroxyphenylacetyl-hexose was significantly enhanced in RA and RC plant with percentages of 1839% and 1454% accordingly. The majority of flavonoid metabolites were found at the highest levels in COM. (i.e., quercetin, quercetin hexose, quercetin 3-glucuronide, quercetin malonylglucoside, quercetin-3-O-(6″-O-crotonyl)-β-glucoside). Two flavanones naringenin 7-neohesperidoside and apigenin 7-Oglucuronide were also highest in COM. Only one flavonoid quercetin-3-O-(6″-O-crotonyl)-β-glucoside) was higher in RA (559%) and RC (1509%) than COM (470%). Some compounds including alangilignoside, coniferoside and eugenol malonylglucoside were significantly increased in RC plants with the percentage of 372%, 357% and 387%, respectively. Among all the putatively identified compounds the greatest change observed was for 2,3,5,4′-tetrahydroxystilbene 2-O-β-D-glucoside which increased 48,008% in RA plants and 40,272% in RC plants.

### 2.6. Unidentified Metabolites

A further 50 unidentified ions-of-interest were shortlisted on the basis that they were altered by shortlisted >1000% in some group. The characteristics of these are tabulated in Table S1.

### 2.7. Antidiabetic Effects of Selected Compounds

**Figure 4:**
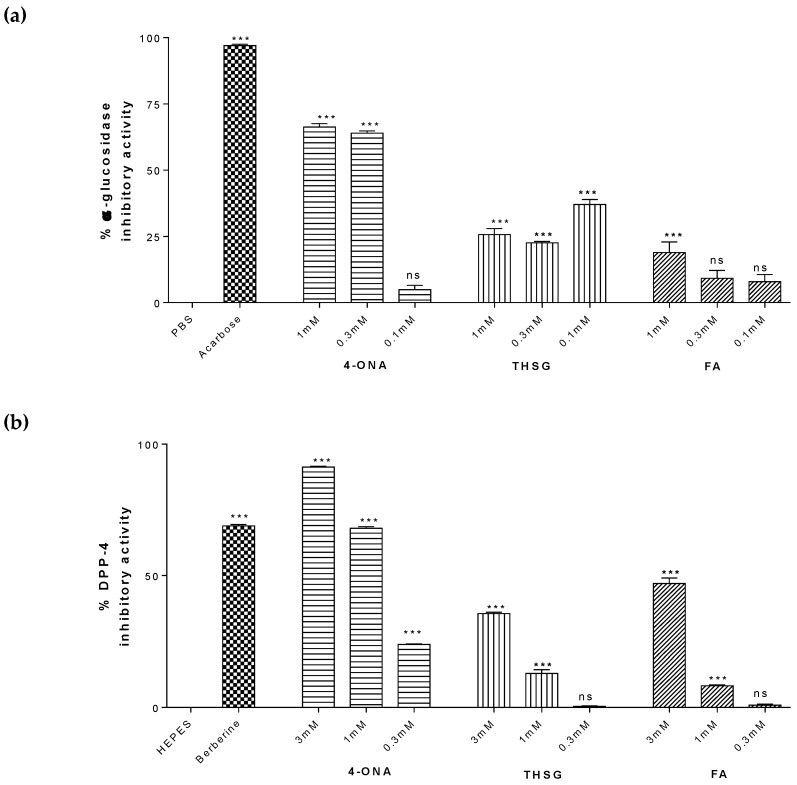

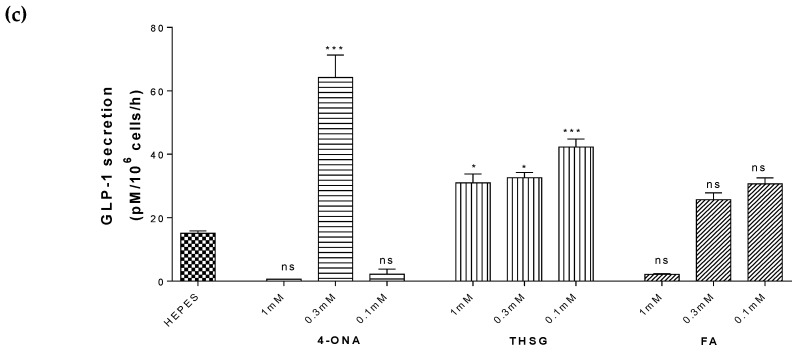
Confirmation of bioactivity of putative plant metabolites identified in *L.sativa*. (**a**) α-glucosidase inhibitory activity of 4-oxononanoic acid (4-ONA), 2,3,5,4′tetrahydroxystilbene-2-O-β-d-glucoside (THSG), and Ferulic acid (FA) (0.1–1.0 mM; *n* = 3). (**b**) Dipeptidyl peptidase-4 (DPP-4) inhibition for, THSG, and FA (0.3–3.0 mM; *n* = 3). (**c**) GLP-1 secretory responses of each ONA, THSG, and FA (0.1–1.0 mM; *n* = 4). All values are mean ± SEM. All statistical comparisons were One Way ANOVA. * *p* < 0.05, ** *p* < 0.01 and *** *p* < 0.001 compared with PBS or HEPES ∆ *p* < 0.05, ∆∆ *p* < 0.01 and ∆∆∆ *p* < 0.001 compared with acarbose or berberine respectively. Inhibitory activities (α-glucosidase and DPP-4) were calculated as a percentage of the vehicle control.

Based on the metabolomics analysis three compounds (2,3,5,4′-tetrahydroxystilbene 2-O-β-d-glucoside (THSG), 4-oxnonanoic acid (4-ONA) and ferulic acid (FA)) identified in *L. sativa* were tested for their ability to inhibit α-glucosidase, inhibit DPP-4 and stimulate secretion of GLP-1. At concentrations of 1 mM and 0.3 mM 4-ONA significantly inhibited α-glucosidase with mean percentage inhibition of 66% (*p* < 0.0001) and 64% (*p* < 0.0001), respectively (Figure 4a). THSG modestly inhibited α-glucosidase at all concentrations tested (23–37%; *p* < 0.0001). FA only inhibited α-glucosidase at 1 mM (19%; *p* < 0.0001). DPP-4 was inhibited by 91.2% ((Figure 4b; *p* < 0.001) by 3 mM 4-ONA which was greater than that of the positive control berberine (69%, *p* < 0.001). At 1 mM and 0.3 mM 4-ONA significantly inhibited DPP-4 by 68% (*p* < 0.001) and of 24% (*p* < 0.001), respectively. THSG and FA showed significant but comparably less inhibition 36% (*p* < 0.001) and 47% (*p* < 0.001). Figure 4c shows the effect of 4-ONA, FA and THSG on the stimulation of GLP-1 secretion from pGIP/Neo cells. Basal secretion (HEPES) levels of GLP-1 were (15.1 ± 0.7 pM/10^6^ cells/h). 4-ONA increased GLP-1 levels approximately 4-fold at 0.3 mM (64.2 ± 7 pM/10^6^ cells/h; *p* < 0.0001). THSG increased levels of GLP-1 secretion by approximately 2-fold at 0.3 mM (32.6 ± 1.6 pM/10^6^ cells/h; *p* < 0.01) and by almost 3-fold at 0.1mM (42.3 ± 2.5 pM/10^6^ cells/h; *p* < 0.0001). FA did not increase GLP-1 secretion at any of the tested concentrations.

## 3. Discussion

There is clear evidence that transformation with individual Agrobacterium *rol* genes overcomes the inability of cultured plants to produce large amounts of secondary metabolites. For example, anthraquinone levels in *rolA* tranforms of *Rubia cordifolia* were increased 2.8-fold [27]; resveratrol levels in *rolB* transforms of grapevine (*Vitus amurensis*) were increased >100-fold [28]; and ginsenoside levels in a *rolC*-transform of *Panax ginseng* were increased 3-fold [5]. Therefore, genetic transformation techniques involving *rol* genes could be an effective tool to manipulate secondary metabolite concentrations in cultured plant cells and alter their properties.

This investigation performed state-of-the-art LC-QTof-MS metabolomic analysis to examine how small molecule metabolites in *L. sativa* are influenced by *rolABC* (RA; a construct of *rolA*, *rolB* and *rolC* genes) and *rolC* (RC). A total of 5333 ions/metabolites were detected and it was possible (by manually searching of metabolomic databases and the published literature) to assign identities to 76 known *L. sativa* metabolites.

The extent to which *rol* genes affected the metabolome of *L.sativa* was extremely broad ranging. Of the 5333 ions quantified the levels of 3637 and 1792, respectively, were significantly changed in RA and RC. This demonstrated that RA and RC had widespread effects on this plant’s metabolism, but interestingly, it also indicated that there were actually more plant metabolites which significantly decreased than increased. Initially this was surprising but perhaps might have been expected—if a plant is focusing its metabolic resources into the synthesis of particular secondary metabolites then this comes at the expense of other metabolic pathways. Furthermore, the Venn diagram (Figure 3a) provides a cursory overview of metabolomic changes, but this does not reveal the specific types of metabolite (i.e., primary or secondary) nor the magnitude by which their concentrations are changed.

The Venn diagram also shows that RA (but not RC) had equally as great an influence on *L.sativa’s* metabolome as the conventional growing environment. Compared with untransformed (UnT) cultured lettuce the levels of 3737 ions/metabolites were significantly different in COM. For RA this is quite impressive given the complexity and substantial influence of the natural environment and also given the potentially large differences in nutrients present in media and soil. As far as the metabolome is concerned *rol* gene manipulation does appear to bridge the gap between tissue culture and agriculture, although the metabolomic profiles of RA and COM are far from being identical. Around 1000 of the changes in metabolite features were not shared by RA and COM, and this is at least one explanation for the differences we observed in their in vitro bioactivity.

Secondary metabolites are frequently associated with various medicinal actions and other forms of bioactivity—many such metabolites have been isolated and their activity characterized. Our ongoing programme of work screening natural products for antidiabetic activity [23,24,25,26,29] indicated that *L.sativa* (cv. Grand Rapids) contained modest levels of inhibitory activity against both α-glucosidase and DPP-4 and also stimulated secretion of the intestinal glucoregulatory hormone GLP-1. A number of secondary plant metabolites have been demonstrated to act on each of these targets. We speculated that bioactivity could serve as a proxy measure for secondary metabolites and hypothesized that *rol* gene manipulation of secondary metabolism (as already indicated by untargeted metabolomics) could lead to greater levels of bioactivity. Indeed, both RA and RC approximately doubled the levels of α-glucosidase and DPP-4 inhibitory activity, and there were many fold increases in their GLP-1 secretory activity. However, for COM the results were not as encouraging. Levels of α-glucosidase inhibitory activity in COM were no different to UnT, and although there was a modest increase in DPP-4 inhibition, GLP-1 secretory activity was actually reduced. This demonstrates that there are specific advantages to be gained from *rol* gene transformation which cannot be gained under conventional growing conditions.

Using the obtained data, it was possible to shortlist 3 of the 76 putatively identified metabolites that were preferentially increased by *rol* gene manipulation. These were: (i) 2,3,5,4′-tetrahydroxystilbene 2-O-β-D-glucoside (THSG) which was increased 40,008% and 40,272% in RA and RC, respectively. This stilbene with strong antioxidant activity and has been shown to improve gastrointestinal motility disorders in streptozotocin (STZ)-induced diabetic mice [30] but does not alter blood glucose levels in this model where insulin is absent. (ii) Ferulic acid methyl ester (FA) which was increased 5616% and 9777% in RA and RC respectively. Ferulic acid is also an antioxidant which has been shown to reduce blood glucose levels in and alleviate peroxidation in STZ-induced diabetic rats [31]. (iii) Amino-oxonanoic acid (OXO) increased 1803% and 1141% in RA and RC, respectively. As yet no effects of amino-oxonanoic acid or related compounds have previously been reported in a diabetes context. We then proceeded to confirm the bioactivity of each of these. Fortunately, THSG and FA were both commercially available whereas for OXO only the closely related compound 4-oxononanoic acid (4-ONA) lacking the amino group could be obtained. These studies found that THSG, FA and 4-ONA possessed significant inhibitory activity against α-glucosidase and DPP-4; and also that THSG and 4-ONA significantly stimulated GLP-1 secretion. There are potentially many other endogenous plant metabolites responsible for the observed changes in plant bioactivity but it is logical to conclude that that THSG, FA and amino-oxonanoic are to some extent responsible for the overall changes in bioactivity levels.

In conclusion, this study performed comparative metabolomic analysis of commercial, *rol ABC* transformed and *rol C* transformed plants, as well as untransformed plants. Our results demonstrate that *rol* gene transformation has a significant and widespread impact on the metabolite composition of lettuce. They also show a significant functional improvement in the plant bioactivity, as determined by three antidiabetic assays which acted as useful surrogate measure of plant secondary metabolism. Most importantly this investigation serves as a new paradigm whereby genetic manipulation, metabolomic and bioactivity techniques can be combined, not only to enable the discovery of novel natural bioactives, but also for us to better understand the dietary significance of plants. 

## 4. Materials and Methods

### 4.1. Plants

Transformed and untransformed plants of *Lactuca sativa* (CV. Grand Rapid) were generated as in our previous study [32,33]. Briefly, seeds were surface sterilized by incubation in ethanol (70%; 1 min), followed by sodium hypochlorite (10%; 45 s), and finally seed were washed 3 times with sterile distilled water. Following sterilization seeds were placed in petri dishes with ½ MS medium under aseptic conditions. Plates were sealed with parafilm and placed in a growth room for 10–14 days (25 ± 2 °C; 16 h photoperiod; illumination: 45 µE m^−2^ s^−1^; 60 % relative humidity). Nodes and cotyledons (0.5^−1^ cm in length) of 7 days old seedlings of in vitro grown lettuce were transformed with Agrobacterium tumefacienes strain GV3101 harbouring either *rolABC* (RA) or *rolC* genes [1]. Kanamycin resistant plant were maintained on Murashige and Skoog (MS) medium [34] supplemented with a filter sterilized solution of α-naphthaleneacetic acid (NAA) (0.1 mg/L) and benzyl aminopurine (BAP) (0.5 mg/L) and were maintained at 25 ± 2 °C (16 h photoperiod; illumination: 45 µE m^−2^ s^−1^; 60 % relative humidity). The transformed plants were selected using kanamycin (50 mg/L) and genetic transformation was confirmed using PCR [32,33]. For comparison purposes the fresh leaves of commercial (COM) *Lactuca sativa* (cv. ‘Grand Rapids’) were purchased from a local market in Rawalpindi (Pakistan) in 2014 with no prior knowledge of their growing conditions. The plant was identified by Dr. Muhammad Zafar (taxonomist) in Plant Sciences Department Quaid-i-Azam University (QAU). A voucher specimen (number 128085) was deposited in the ‘Herbarium of medicinal Plants of Pakistan’ in QAU Islamabad, Pakistan.

### 4.2. Extraction of Plant Material

*Lactuca sativa* leaves were washed with distilled water and were air dried under shade for five weeks at room temperature. The leaves were then ground to powder and underwent successive solvent extraction in gradient of increasing solvent polarity. Briefly, dried plant powder (1 g) was macerated overnight at 20 ± 5 °C in n-hexane, chloroform, methanol and water in a successive manner. For each solvent (20 mL) there were three rinses and the resulting solution was vortexed (5 min) and sonicated (5 min). Extracts were then filtered (Whatman #1) and the filtrate dried under vacuum (40 °C). The hexane, chloroform, methanol and aqueous extracts were stored at −20 °C. After a series of trial metabolomic and bioactivity experiments only the methanol extracts were taken forward for analysis. This was based on: (i) that hexane and chloroform extracts were devoid of bioactivity and were cytotoxic in cell studies, and (ii) that the aqueous extract (despite containing bioactivity) had poor ionization properties when subjected to LC-qTof-MS in both positive and negative modes of acquisition.

### 4.3. UPLC-QTof-MS Analysis of Lactuca Sativa

Chromatography was performed on a Waters Acquity UPLC I-Class system (Milford, MA, USA) equipped with column oven, coupled to a Waters Xevo G2-XS QTof mass spectrometer (Manchester, UK) equipped with an electrospray ionisation source operating in both positive and negative mode with lock-spray interface for real time accurate mass correction. The source temperature was 120 °C with a cone gas flow of 25 L/h, a desolvation temperature of 450 °C, and a desolvation gas flow of 850 L/h. The capillary voltage was set at 0.6 kV for HILIC analysis and 1.0 kV for Reverse phase analysis with a cone voltage of 30 V. A lock-mass solution of Leucine Enkephalin (2 ng·µL^−1^) in acetonitrile/water containing 0.1% formic acid (50:50, *v*/*v*) was continuously infused into the MS via the lock-spray at a flow rate of 5 µL·min^−1^.

A 2.5 μL aliquot of methanolic extract (5 mg/mL reconstituted in HPLC-grade water) was injected onto an Acquity Cortecst UPLCr C18 1.6 µm (Waters, Milford, MA, USA). The column oven was set at 50 °C and the sample manager temperature was 10 °C. The gradient elution buffers were A (5 mM ammonium formate) and B (acetonitrile containing 0.025% formic acid), and the flow rate was 0.6 mL·min^−1^. The elution gradient (A:B, *v*/*v*) was as follows: an isocratic period of 2 min at 5:95 followed by a linear gradient from 5:95 to 30:70 over 8 min then a linear change from 30:70 to 90:10 over 1 min. After a 1 min period at 90:10, a linear gradient was applied over 0.5 min to return to the initial composition 5:95 which was held for 3.5 min before the next injection.

Prior to all analyses eight pooled conditioning samples were injected. Before the analysis eight pooled acclimatizing samples were injected. For quality control pooled samples were injected at intervals every eight samples throughout the entire experiment to determine the chromatographic reproducibility of retention times and peak intensities [35]. All LC-MS solvents used were purchased from Sigma-Aldrich (Dorset, UK) and were LC-MS grade or equivalent.

### 4.4. Data Analysis

The raw data from the spectral analysis of the *Lactuca sativa* extracts was processed using Progenesis QI v2.0 Software (Waters Corporation, Milford, USA). Progenesis QI enables the accurate processing of high resolution LC-MS spectral data whilst also providing annotation of putative compounds based on accurate mass measurements and fragmentation information. This software aids in both the validation of LC-MS approaches and identification of features within the spectral data. Using Progenesis QI the spectral data were aligned to a chosen pooled sample, adduct ions were deconvoluted and ions abundance of features above the threshold level calculated.

Following analysis in Progenesis QI, the raw data were filtered to remove any variables containing >20% zero values (considered technical noise). The data were exported to Simca 13 (Umetrics, Umea, Sweden) for multivariate analysis. For quality control purposes all the spectral data were Center-Scaled and analyzed using principal components analysis (PCA). All pooled samples were found to be tightly clustered within the center of each representative scores plot indicating that the methodology had extremely good technical reproducibility [35]. Subsequently all data were mean centered and Pareto scaled, and then divided into four groups UnT (untransformed), RA (*rolABC* transformed), RC (*rolC* transformed) and COM (commercially obtained), prior to analysis by Orthogonal Projections to Latent-Discriminant Analysis (OPLS-DA). Predictive powers were based on the Q2 score produced using Simca P. This involved the data being randomly divided into seven parts (by default) and during each analysis cycle 1/7th in turn is removed. A model was built on the 6/7th data left in and the removed data were predicted from the new model. This was repeated with each 1/7th of the data until all the data had been classified. The predicted data were then compared with the original data and the Predicted Residual Sum of Squares (PRESS) was calculated for the whole dataset. Q2 is calculated from the PRESS value which was divided by the initial sum of squares and subtracted from one. Models with good predictive powers have low PRESS scores and high Q2 values.

### 4.5. Plant Metabolite Identification

The identification of metabolites was conducted manually using MassLynx 4.1 software (Waters, Milford, MA, USA) which has tools for examining elemental composition, isotope patterns, theoretical monoisotopic, nominal and average masses. Firstly, the likely elemental composition of each candidate compound was determined by generating a list of possible molecular formulae based on a CHNO (carbon, hydrogen, nitrogen, oxygen) algorithm. MassLynx offers different functionalities such as elemental range, electron configuration, and double-bond equivalents, the deviation error between the theoretical and measured mass in terms of Da and ppm, and provides sigma values by comparing theoretical isotope patterns with the measured ones. The broadly accepted error values of Da and ppm was set at 50 and 5, respectively. The molecular formula, monoisotopic masses, chromatogram, their MS spectrum and their fragmentation pattern were compared against *L. sativa* literature and online available databases. Metlin (http://metlin.scripps.edu), HMDB (http://www.hmdb.ca) and MassBank (http://www.massbank.jp) were used to identify the fragmentation patterns of putative ions. other databases were also consulted to confirm classification, pathways and their biological roles e.g, Kegg Ligand Database (http://www.genome.jp/kegg/ligand.html), Phenol-Explorer (www.phenol-explorer.eu), PubChem (http://pubchem.ncbi.nlm.nih.gov), ChemSpider (http://www.chemspider.com). The percentage change was calculated for each identified metabolite. Differences determined by one-way ANOVA with Bonferroni correction and data were deemed statistically significant if *p* < 0.05.

### 4.6. Measurement of Alpha-Glucosidase Inhibition

Alpha-glucosidase inhibition was assessed by monitoring the rate of glucose formation from carbohydrates incubated with rat intestinal extract (Sigma, Dorset, UK) in the presence of each sample. Reaction mixtures contained 300 µL of maltose (10 mg/mL) and 150 µL of either PBS (pH 7.4, as a negative control), acarbose (30 mg/mL, as a positive control), or compound (1 mM, 0.3 mM and 0.1 mM). These were pre-incubated in a water bath (37 °C; 10 min) and reactions were initiated by the addition of rat intestinal acetone extract. The optimal amount of intestinal extract required for maltose incubations was determined by a series of preliminary experiments. Glucose concentrations were measured at 0, 30, 60 and 90 min incubation time points using an enzymatic assay kit (Analox Ltd. London, UK) and detected on a PGM7 Micro-Stat Analyser (Analox Instruments Ltd.; London, UK).

### 4.7. Measurement of DPP-4 Inhibitory Activity

DPP-4 inhibitory activity was determined using a previously described method [36] where free AMC (7-amino-4-methyl-coumarin) is liberated from DPP-4 substrate Gly-Pro-AMC. Briefly, samples were prepared in PBS (pH 7.4) and assays were conducted in triplicate using a 96 well microtiter plate. Each well contained 20 µL of sample (3 mM, 1 mM and 0.3 mM), 20 µL of porcine DPP-4 (1 U/mL) (EMD Millipore, UK) and 30 µL of 1 mM Gly-Pro-AMC (Bachem, Switzerland). Plates were incubated (37 °C; 1 h) with gentle agitation. Reactions were stopped by addition of acetic acid (100 µL; 3 mM) and fluorescence (Ex 351 nm/Em 430 nm) measured (Safire II fluorometer, Tecan, Reading, UK). Berberine an isoquinoline alkaloid with DPP-4 inhibitory activity (IC50 13.3 µM) was used as a positive control in each experiment.

### 4.8. GLP-1 Secretory Responses in pGIP/Neo STC-1 Cells

pGIP/Neo STC-1 cells were a gift from Dr. B. Wice (Washington University of St. Louis) (Ramshur, Rull & Wice, 2002) with permission from Dr D. Hanahan (University of California, San Francisco, CA). Culture medium contained DMEM containing 4.5 g/L with l-glutamine, without sodium pyruvate (Life Technologies, Paisley, UK) and supplemented with 10% foetal bovine serum, 100 U/mL penicillin, 100 mg/L streptomycin and geneticin (G418; 400 μg/mL; Sigma, Dorset, UK). pGIP/Neo STC-1 cells were cultured in culture medium and incubated in a 5% CO_2_ humidified atmosphere at 37 °C. Cells underwent passage upon reaching 80–90% confluence and were used between passage numbers 15–50.

For secretion studies cells were seeded into 12-well plates (2 × 106 per well) and cultured overnight at 37 °C in a humidified atmosphere of 5% CO_2_. Medium was removed and cells washed (×3) with HEPES buffer (20 mM HEPES, 10 mM glucose, 140 nM NaCl, 4.5 mM KCl, 1.2 mM CaCl_2_, 1.2 mM MgCl_2_) then allowed to incubate for 1 h in HEPES buffer prior to adding fresh HEPES buffer (vehicle control) or HEPES buffer supplemented with test agents (1 mM, 0.3 mM and 0.1 mM). Cells were incubated for 3 h after which the vehicle control and test agents were removed, centrifuged and stored at −80 °C prior to ELISA assays. GLP-1 ELISA kits (detecting only active GLP-1 forms (7–36 amide) and (7–37)) were purchased from Millipore (Billerica, MA, USA).

### 4.9. Data Analysis of Bioactivity Studies

All values from bioactivity studies were expressed as mean ± standard error mean (SEM). Percentage α-glucosidase inhibition was calculated by measuring area under curve (AUC) and expressing this as a percentage of the negative control. Data were analysed by one-way ANOVA with Tukey’s multiple comparison test (GraphPad Prism, v5.0, San Diego, CA, USA) with data deemed significant if *p* < 0.05.

## Figures and Tables

**Table 1:** Changes in the levels of sesquiterpene lactones in *Lactuca sativa.*

No.	Compound	*Rol* ABC (RA)			*Rol* C (RC)			Commercial (COM)		
		↑/↓	% Change ± SEM	*p* Value	↑/↓	% Change ± SEM	*p* Value	↑/↓	% Change ± SEM	*P* Value
1	Lactucopicrin	↑	17 ± 10.3	0.272	↑	85 ± 31.6	0.048 *	↑	90 ± 8.9	0.009 **
2	Lactucopicrin-15-oxalate	↓	79 ± 14.1	0.020 *	↑	29 ± 18.3	0.183	↓	6 ± 0.5	0.403
3	11β,13-dihydrolactucopicrin	↑	132 ± 6.3	0.002 **	↑	257 ± 4.4	0.006 **	↑	127 ± 4.9	0.003 **
4	Lactucin	-	Unchanged	N/A	↑	unchanged	N/A	↑	667 ± 145.3	0.005 **
5	Lactucin-sulfate	↑	25 ± 5.6	0.177	↑	95 ± 26.7	0.027 *	↑	90 ± 11.1	0.009 **
6	15-deoxylactucin-8-sulfate	↓	93 ± 3.1	0.008 **	↓	95 ± 1.5	0.007 **	↓	56 ± 1.0	0.035 *
7	15-deoxylactucin-8-sulfate-Gln	↑	535 ± 98.9	0.005 **	↑	511 ± 68.8	0.001 **	↓	89 ± 5.3	0.009 *
8	15-deoxylactucin-8-sulfate-Pro	↓	96 ± 1.4	0.007 **	↓	97 ± 0.4	0.007 **	↑	50 ± 1.8	0.048 **
9	15-deoxylactucin-8-sulfate-Val	↑	1103 ± 99.1	0.061	↑	1028 ±164.9	0.002 **	↓	20 ± 3.1	0.214
10	8-deacetylmatricarin-8-sulfate	↑	28 ± 15.8	0.182	↑	128 ± 43.8	0.030 *	↑	207 ± 13.1	0.001 **
11	Cichorioside B-sulfate	↑	83 ± 10.3	0.014 *	↑	149 ± 26.9	0.006 *	↑	52 ± 8.6	0.047 *
12	1-β-(4-hydroxyphenylacetyl)-15-O-β-d-glucopyranosyl-5α, 6βH-eudesma-3-en-12, 6α-olide	↑	327 ± 48.2	0.029 *	↓	29 ± 23.6	0.220	↓	100 ± 9.8	0.007 **

Table shows the percentage change in levels of sesquiterpene lactones compared with untransformed (unT) *Lactuca sativa* plants. Values are % mean (± SEM) and P values indicate differences with unT plants (* *p* < 0.05, ** *p* < 0.01).

**Table 2:** Changes in the levels of non-phenolic metabolites in *Lactuca sativa.*

No.	Compound	*Rol* ABC (RA)			*Rol* C (RC)			Commercial (COM)		
		↑/↓	% Change ± SEM	*p* Value	↑/↓	% Change ± SEM	*p* Value	↑/↓	% Change ± SEM	*p* Value
1	Leucine/Isoleucine	↑	168 ± 19.5	0.003 **	↑	457 ± 67.2	0.002 **	↑	794 ± 7.9	0.001 **
2	Glycine-Leucine	↑	57 ± 36.6	0.126	↑	69 ± 11.9	0.024 *	↑	289 ± 5.4	0.001 **
3	Glycine-Leu/Ileu-hexose	↑	2192 ± 659.5	0.015 *	↑	2039 ± 960	0.050 *	↑	2292 ± 38.7	0.001 **
4	Tryptophan	↓	22 ± 18.4	0.241	↑	107 ± 65.1	0.096	↑	494 ± 9.8	0.001 **
5	Tryptophan-hexose	↑	523 ± 193.4	0.031 *	↑	516 ± 277.1	0.071	↑	1514 ± 664.6	0.043 *
6	Tyrosine	↑	160 ± 32.9	0.007 **	↑	401 ± 76.0	0.004 **	↑	282 ± 2.9	0.001 **
7	Tyrosine-hexose	↑	688 ± 164.9	0.007 **	↑	1295 ± 41.9	0.001 **	↑	2897 ± 78.9	0.001 **
8	Phenylalanine	↑	168 ± 35.5	0.008 **	↑	540 ± 148.3	0.011 *	↑	2968 ± 13.5	0.001 **
9	Phenylalanine-hexose	↑	95 ± 16.4	0.012 *	↑	206 ± 1.8	0.001 **	↑	2567 ± 20.3	0.001 **
10	Malic acid	↓	100 ± 0.9	0.005 **	↓	100 ± 0.9	0.005 **	↓	51 ± 3.0	0.038 *
11	Citramalic acid	↓	38 ± 5.8	0.080	↓	29 ± 4.7	0.126	↑	59 ± 20.3	0.059
12	Pyroglutamic acid	↑	53 ± 10.3	0.047 *	↑	154 ± 22.8	0.004 **	↑	51 ± 2.2	0.042 *
13	Pyroglutamic acid-Leucine/Isoleucine	↑	152 ± 60.2	0.038 *	↑	294 ± 77.8	0.011 *	↑	87 ± 0.8	0.008 **
14	Uridine	↑	108 ± 44.0	0.047 *	↑	207 ± 20.5	0.001 **	↑	126 ± 3.2	0.002 **
15	Adenosine	↑	84 ± 58.1	0.124	↑	148 ± 27.3	0.007 **	↑	224 ± 1.9	0.001 **
16	Guanosine	↑	200 ± 67.7	0.024 *	↑	255 ± 54.4	0.006 **	↑	113 ± 0.2	0.003 **
17	Isopropylmalic acid	↑	56 ± 7.8	0.038 *	↑	43 ± 45.7	0.223	↑	537 ± 19.3	0.001 **
18	1,2,3,4-Tetrahydro-beta-carboline-3-carboxylic acid	↓	50 ± 9.5	0.071	↑	125 ± 96.4	0.139	↑	1698 ± 13.2	0.001 **
19	1,2,3,4-Tetrahydro-b-carboline-1,3-dicarboxylic acid	↓	38 ± 6.6	0.081	↑	147 ± 90.3	0.094	↑	1567 ± 27.7	0.001 **
20	Azelaic acid	↓	66 ± 4.8	0.022 *	↑	52 ± 52.6	0.208	↓	26 ± 1.4	0.155
21	Quinic acid	↓	61 ± 3.5	0.026 *	↓	46 ± 10.3	0.065	↓	72 ± 0.4	0.016 *
22	Citric Acid	↑	169 ± 26.6	0.004 **	↑	508 ± 137.3	0.011 *	↑	1137 ± 241.7	0.005 **
23	Amino oxononanoic acid	↑	1803 ± 388.3	0.005 **	↑	1141 ± 337.1	0.015 *	↑	188 ± 41.5	0.030 *
24	Pantothenic acid (vitamin B5)	↑	128 ± 10.0	0.003 **	↑	79 ± 167.3	0.332	↓	100 ± 0.9	0.005 **

Table shows the percentage change in levels of non-phenolic metabolites compared with untransformed (unT) *Lactuca sativa* plants. Values are % mean (± SEM) and P values indicate differences with unT plants (* *p* < 0.05, ** *p* < 0.01).

**Table 3:** Changes in the levels of phenolic metabolites in *Lactuca sativa.*

No.	Compound	*Rol* ABC (RA)			*Rol* C (RC)			Commercial (COM)		
		↑/↓	% Change ± SEM	*p* Value	↑/↓	% Change ± SEM	*p* Value	↑/↓	% Change ± SEM	*p* Value
1	Hydroxybenzoic acid	↓	25 ± 14.9	0.170	↑	71 ± 35.2	0.074	↓	70 ± 4.0	0.009 **
2	Dihydroxybenzoic acid	↓	59 ± 5.5	0.017 *	↑	48 ± 50.8	0.213	↑	223 ± 53.1	0.008 **
3	3-Methoxy-4-hydroxyphenyl-1-O-β-d-glucoside	↑	115 ± 47.9	0.044 *	↑	153 ± 26.1	0.004 **	↑	28 ± 17.7	0.163
4	Syringic acid	↑	Unchanged	N/A	↑	unchanged	N/A	↑	unchanged	N/A
5	Syringic acid hexose	↓	49 ± 1.1	0.027 *	↑	35 ± 33.5	0.203	↓	76 ± 3.4	0.007 **
6	Vanillic Acid	↑	Unchanged	N/A	↑	unchanged	N/A	↑	unchanged	N/A
7	Vanillic acid glucoside	↓	51 ± 11.5	0.037 *	↓	44 ± 8.2	0.046 *	↓	10 ± 14.5	0.340
8	Dihydroxybenzoic acid hexose	↓	77 ± 1.2	0.007 **	↓	25 ± 19.8	0.205	↑	66 ± 23.1	0.044 *
9	Hydroxybenzoyl dihydroxybenzoyl-hexose	↓	35 ± 18.5	0.123	↑	44 ± 35.6	0.166	↑	36 ± 18.8	0.121
10	Caffeic acid	↓	3 ± 9.9	0.439	↑	131 ± 35.1	0.015 *	↑	326 ± 59.1	0.003 **
11	Dihydrocaffeic acid hexose	↑	65 ± 40.0	0.105	↑	56 ± 17.8	0.047 *	↓	9 ± 12.3	0.344
12	Caffeoyl-hexose	↑	55 ± 15.5	0.040 *	↑	62 ± 10.1	0.020 *	↑	69 ± 23.2	0.039 *
13	Ferulic acid	↓	34 ± 10.2	0.089	↑	20 ± 33.2	0.310 *	↑	38 ± 19.3	0.114
14	Ferulic acid glucoside	↓	71 ± 3.9	0.009 **	↓	49 ± 10.1	0.038 *	↑	8 ± 14.9	0.376
15	Ferulic acid methyl ester	↑	5616 ± 231	0.036 *	↑	9777 ± 291	0.014 *	↑	3033 ± 458	0.001 **
16	Sinapoyl glucoside	↑	21 ± 11.8	0.193	↑	41 ± 54.9	0.259	↑	230 ± 46.1	0.005 **
17	p-Coumaroyl glucoside	↓	46 ± 5.6	0.036 *	↓	28 ± 6.2	0.107	↑	880 ± 140.3	0.002 **
18	Caftaric acid	↓	80 ± 8.4	0.008 **	↓	91 ± 2.3	0.004 **	↑	2290 ± 332	0.001 **
19	Tartaric acid	↑	Unchanged	N/A	↑	unchanged	N/A	↑	unchanged	N/A
20	Coutaric acid	↓	92 ± 2.4	0.004 **	↓	83 ± 6.4	0.006 **	↑	69 ± 24.0	0.042 *
21	Caffeoyltartaric-p-coumaroyl acid	↓	87 ± 4.6	0.005 **	↓	73 ± 11.8	0.014 *	↑	822 ± 134.9	0.002 **
22	Caffeoylquinic acid	↓	39 ± 5.9	0.055	↓	11 ± 13.2	0.322	↓	78 ± 3.0	0.006 **
23	Dicaffeoylquinic acid	↓	75 ± 6.2	0.009 **	↓	61 ± 10.4	0.021 *	↓	94 ± 0.9	0.003 **
24	p-Coumaroyl-caffeoylquinic acid	↓	5 ± 17.2	0.425	↓	52 ± 6.7	0.027 *	↓	86 ± 2.0	0.004 **
25	p-Coumaroylquinic acid	↓	75 ± 9.1	0.011 *	↓	18 ± 4.5	0.196	↑	3978 ± 572	0.001 **
26	4-hydroxyphenylacetyl glucoside	↑	119 ± 27.4	0.011	↑	40 ± 102.7	0.360	↓	65 ± 6.0	0.013 *
27	Di(4-hydroxyphenylacetyl)-hexose	↑	1839 ± 438	0.007 **	↑	1454 ± 114	0.136	↓	86 ± 3.4	0.005 **
28	Quercetin	↓	26 ± 45.6	0.314	↓	73 ± 18.3	0.023 *	↑	3952 ± 672	0.002 **
29	Quercetin hexose	↓	69 ± 3.2	0.011 *	↓	38 ± 27.6	0.155	↑	12,022 ± 173	0.001 **
30	Quercetin 3-glucuronide	↓	78 ± 12.0	0.012 *	↓	82 ± 7.4	0.007 **	↑	44,309 ± 6178	0.001 **
31	Quercetin malonylglucoside	↓	91 ± 3.2	0.004 **	↓	92 ± 1.2	0.004 **	↑	361 ± 65.3	0.003 **
32	Quercetin-3-O-(6″-O-crotonyl)-β-glucoside	↑	559 ± 59.9	0.001 **	↑	1509 ±319	0.005 **	↑	470 ± 78.7	0.002 **
33	Naringenin 7-neohesperidoside	↓	51 ± 7.7	0.030 *	↓	27 ± 10.9	0.133	↓	81 ± 2.5	0.006 **
34	Apigenin 7-O-glucuronide	↑	199 ± 76.5	0.032 *	↑	81 ± 57.3	0.124	↑	3740 ± 531	0.001 **
35	Syringaresinol-β-d-glucoside	↓	43 ± 1.4	0.039 *	↓	7 ± 10.9	0.382	↓	55 ± 6.5	0.023 *
36	Syringaresinol malonylhexose	↓	95 ± 1.1	0.003 **	↓	97 ± 0.4	0.003 *	↓	100 ± 0.0	0.003 **
37	Alangilignoside C	↑	224 ± 22.3	0.001 **	↑	372 ± 110.2	0.015 *	↓	99 ± 0.2	0.003 **
38	Coniferoside	↑	111 ± 64.1	0.085 *	↑	357 ± 154.5	0.042 *	↑	272 ± 51.5	0.004 **
39	2,3,5,4β-Tetrahydroxystilbene2-O-β-D-glucoside	↑	48,008 ± 365	0.001 **	↑	40,272 ± 1204	0.014 *	↑	11,944 ± 207	0.002 **
40	Eugenol malonylglucoside	↑	171 ± 78.7	0.051	↑	387 ± 88.6	0.006 **	↓	92 ± 1.6	0.004 **

Table shows the percentage change in levels of phenolic metabolites compared with untransformed (unT) *Lactuca sativa* plants. Values are % mean (± SEM) and P values indicate differences with unT plants (* *p* < 0.05, ** *p* < 0.01).

## Supplementary Materials

The following are available online at https://www.mdpi.com/2218-1989/9/5/97/s1, Table S1: Changes in the levels of some unidentified metabolites in *Lactuca sativa*, Table S2: LC-MS characteristics of sesquiterpene lactone metabolites putatively identified in *Lactuca sativa*, Table S3: LC-MS characteristics of non-phenolic metabolites putatively identified in *Lactuca sativa*, Table S4: LC-MS characteristics of phenolic metabolites putatively identified in *Lactuca sativa.*
