# Supplementary material for: The Health Promoting Bioactivities of Lactuca sativa can be Enhanced by Genetic Modulation of Plant Secondary Metabolites
Source: Metabolites. 2019 May 12;9(5):97. doi: 10.3390/metabo9050097 (PMC6572300; doi:10.3390/metabo9050097)
Supplement: Supplementary file 1 [file metabolites-09-00097-s001.pdf]

metabolites

MDPI

Article

SUPPLMENTARY DATA: "The health promoting bioactivities of Lactuca sativa can be enhanced by genetic modulation of plant secondary metabolites."

Hamad Ismail[1], Anna L Gillespie[2], Danielle Calderwood[2], Haroon Iqbal[2], Colene Gallagher[2], Olivier P Chevallier[2,3], Christopher T Elliott[2,3], Xiaobei Pan[2], Bushra Mirza[1], Brian D Green[2,3]*

[1] Department of Biochemistry, Quaid-I-Azam University, 45320, Islamabad, Pakistan.
[2] Institute for Global Food Security, School of Biological Sciences, Queen's University Belfast, Chlorine Gardens, Belfast, BT9 5DJ, UK;
[3] Core Technology Unit for Mass Spectrometry, Faculty of Medicine, Health and Life Sciences, Queen's University Belfast, UK
* Correspondence: b.green@qub.ac.uk; Tel.: +44-2890-97-6541.

Supplementary Data contained in this document: Table S1: Changes in the levels of some unidentified metabolites in *Lactuca sativa*, Table S2: LC-MS characteristics of sesquiterpene lactone metabolites putatively identified in *Lactuca sativa*, Table S3: LC-MS characteristics of non-phenolic metabolites putatively identified in *Lactuca sativa*, Table S4: LC-MS characteristics of phenolic metabolites putatively identified in *Lactuca sativa.*

Table S1 Changes in the levels of some unidentified metabolites in *Lactuca sativa*

No.	Retention time	Detected mass	Rol ABC (RA)			Rol C (RC)			Commercial (COM)		
			↑/↓	% Change ± SEM	P value	↑/↓	% Change ± SEM	P value	↑/↓	% Change ± SEM	P value
1	7.18	462.0791[ɣ]	↓	94 ± 4.9	0.001**	↓	96 ± 3.0	0.001**	↑	33,528 ± 138.9	0.001**
2	7.41	464.0949 [ɣ]	↓	92 ± 1.6	0.001**	↓	85 ± 5.0	0.001**	↑	5,067 ± 73.3	0.001**
3	7.30	448.0999 [ɣ]	↓	91 ± 3.4	0.001**	↓	55 ± 21.6	0.073	↑	3,246 ± 5.5	0.001**
4	9.18	286.0464 [ɣ]	↓	54 ± 25.3	0.099	↓	89 ± 2.7	0.001**	↑	5,251 ± 226.7	0.001**
5	10.12	375.2016	↓	80 ± 3.6	0.001**	↓	52 ± 3.2	0.004**	↑	6,594 ± 260.0	0.001**
6	9.42	423.1648	↑	29 ± 36.8	0.161	↑	57 ± 49.3	0.114	↑	35,741 ± 162.5	0.001**
7	13.89	577.2664	↑	594 ± 220.4	0.026*	↓	56 ± 29.7	0.119	↑	1,515 ± 47.8	0.001**
8	1.65	585.2865	↑	1,193 ± 223.1	0.003**	↑	2,710 ± 471.1	0.002**	↑	6,537 ± 124.1	0.001**

9	8.57	415.1031	↑	916 ± 423.5	0.047*	↑	592 ± 196.4	0.018*	↑	5,704 ± 146.6	0.001**
10	8.74	208.0723 ɣ	↑	315 ± 150.8	0.047*	↑	257 ± 104.6	0.026*	↑	7,473 ± 217.5	0.001**
11	12.88	311.2212	↑	408 ± 186.7	0.044*	↑	358 ± 70.5	0.003**	↑	5,137 ± 2.9	0.001**
12	3.10	327.1304 ɣ	↑	87 ± 21.3	0.005**	↑	327 ± 92.8	0.011*	↑	2,759 ± 37.2	0.001**
13	6.79	307.1384	↓	34 ± 18.7	0.188	↓	22 ± 6.3	0.217	↑	1,106 ± 11.7	0.001**
14	5.43	254.0787 ɣ	↑	71 ± 26.0	0.018*	↑	265 ± 81.9	0.014*	↑	2,124 ± 75.5	0.001**
15	13.36	313.2367	↑	1,149± 577.8	0.057	↑	729 ± 102.2	0.001**	↑	1,349 ± 47.6	0.001**
16	3.48	294.0940 ɣ	↑	1,431 ± 178.9	0.034*	↑	1,635 ± 140.0	0.005**	↓	99 ± 1.8	0.001**
17	13.74	675.3009	↑	1,771 ± 622.5	0.023*	↓	11 ± 29.7	0.463	↓	60 ± 4.0	0.001**
18	13.94	675.3000	↑	1,841 ± 328.2	0.030*	↓	51 ± 16.5	0.052	↓	86 ± 1.7	0.001**
19	14.73	555.2831	↑	1,182 ± 598.6	0.058	↓	64 ± 30.6	0.094	↑	499 ± 30.8	0.001**
20	13.43	649.2848	↑	2,030 ± 736.9	0.025*	↓	35 ± 44.6	0.330	↓	94 ± 2.3	0.001**
21	14.77	571.2875	↑	1,462 ± 497.0	0.021*	↓	53 ± 41.6	0.201	↑	63 ± 9.6	0.001**
22	14.68	705.3471	↑	1,731 ± 451.5	0.009**	↓	31 ± 31.6	0.119	↓	72 ± 6.5	0.001**
23	13.82	663.3005	↑	1,287 ± 397.7	0.015*	↓	31 ± 34.6	0.321	↓	90 ± 1.9	0.001**
24	14.44	691.3315	↑	1,227 ± 68.4	0.013*	↓	66 ± 8.2	0.004*	↓	95 ± 1.9	0.001**
25	14.34	722.3013	↑	1,653 ± 265.0	0.033*	↓	13 ± 22.4	0.488	↓	42 ± 4.7	0.009**
26	14.93	438.2720 ɣ	↑	2,995 ± 932.1	0.016*	↑	123 ± 142.5	0.196	↑	384 ± 11.3	0.001**
27	14.22	293.2101	↑	1,433 ± 442.7	0.015*	↑	394 ± 149.8	0.027*	↑	63 ± 17.2	0.001**
28	14.44	1017.5232	↑	1,722 ± 121.0	0.002**	↑	32 ± 33.9	0.128	↓	73 ± 4.4	0.001**
29	2.30	370.1456 ɣ	↑	1,318 ± 127.8	0.030*	↑	1,405 ± 58.9	0.001**	↓	67 ± 1.8	0.001**
30	2.51	415.1441	↑	1,475 ± 149.2	0.001**	↑	1,185 ± 354.5	0.014*	↑	15 ± 3.2	0.009**
31	14.12	293.2101	↑	1,979± 338.0	0.021*	↑	1,134 ± 20.4	0.001**	↑	115 ± 12.2	0.001**
32	13.68	293.2104	↑	1,221 ± 127.3	0.070	↓	28 ± 7.6	0.116	↑	748 ± 56.2	0.001**
33	14.65	675.3381	↑	1,399 ± 81.9	0.004*	↓	17 ± 5.6	0.365	↓	43 ± 2.2	0.008**

34	14.22	689.3231	↑	1,345 ± 105.5	0.014*	↓	4 ± 4.1	0.163	↓	32 ± 1.8	0.031*
35	14.14	295.2255	↑	1,250 ± 96.7	0.026*	↑	5 ± 13.6	0.151	↑	237 ± 24.4	0.001**
36	0.96	290.1034	↑	96 ± 108.2	0.184	↑	3,271 ± 110.5	0.175	↑	3,531 ± 444.2	0.075
37	0.96	581.2152	↑	195 ± 183.9	0.160	↑	5,350 ± 541.5	0.174	↑	5,179 ± 585.9	0.061
38	4.70	706.2374	↑	154 ± 67.8	0.035*	↑	1,205 ± 24.5	0.001*	↓	99 ± 1.8	0.001**
39	9.45	412.2070 ɣ	↓	19 ± 4.3	0.283	↑	1,134 ± 22.0	0.054	↓	94 ± 1.9	0.001**
40	6.65	424.1742 ɣ	↓	10 ± 28.7	0.458	↑	1,135 ± 29.3	0.092	↓	92 ± 1.8	0.001**
41	9.14	455.1916	↓	9 ± 14.7	0.392	↑	1,149 ± 16.5	0.013*	↓	92 ± 1.9	0.001**
42	2.89	370.1457 ɣ	↑	372 ± 137.2	0.024*	↑	1,451 ± 63.9	0.001**	↓	57 ± 1.8	0.002**
43	6.11	369.1180	↑	34 ± 29.1	0.093	↑	1,133 ± 27.3	0.003**	↓	70 ± 2.8	0.001**
44	6.46	471.1871	↓	20 ± 2.5	0.213	↑	1,120 ± 6.8	0.014*	↓	90 ± 1.8	0.001**
45	1.58	638.1980	↓	154 ± 31.1	0.003*	↑	1,467 ± 109.7	0.006**	↑	1,012 ± 5.0	0.001**
46	10.91	327.2167	↑	10 ± 9.5	0.396	↑	1,216 ± 101.2	0.043*	↑	570 ± 6.8	0.001**
47	11.39	330.2394 ɣ	↓	17 ± 20.3	0.116	↑	1,272 ± 129.6	0.046*	↑	117 ± 2.1	0.001**
48	1.58	292.1391	↑	57 ± 11.9	0.004*	↑	1,204 ± 46.9	0.005**	↑	474 ± 7.2	0.001**
49	11.63	329.2319	↑	66 ± 12.5	0.003*	↑	1,299 ± 93.2	0.014*	↑	64 ± 1.9	0.001**
50	6.83	369.1180	↑	104 ± 33.0	0.013*	↑	1,223 ± 32.3	0.001**	↓	6 ± 2.1	0.170

Table shows the percentage change in levels of unidentified metabolites compared with untransformed (unT) *Lactuca sativa* plants. Values are % mean (± SEM) and P values indicate differences with unT plants (* $p < 0.05$, ** $p < 0.01$). Detected masses were represented as m/z $[M-H]^-$ and "n" represents the neutral mass

Table S2 LC-MS characteristics of sesquiterpene lactone metabolites putatively identified in *Lactuca sativa*

**No.**	**RT (min)**	**Observed m/z ([M-H]⁻)**	**Calculated m/z ([M-H]⁻)**	**Error (ppm)**	**Major Fragments m/z ([M-H]⁻)**	**Putative compound**	**Empirical formula**	**Empirical weight**	**Class**
1	2.97	409.1297	409.1287	2.4	257.0753, 213.0968, 151.0391, 107.0781	Lactucopicrin	C23H22O7	410.4166*	Lactone
2	13.97	481.1233	481.1135	20.4	257.0885, 213.0759, 151.0316, 107.044	Lactucopicrin-15-oxalate	C25H22O10	482.1140	Lactone
3	5.62	411.1485	411.1444	10.0	277.1069, 259.0780, 215.1066, 151.0391, 107.0399	11β,13-dihydrolactucopicrin	C23H24O7	412.1449	Lactone
4	6.95	275.0934	275.0919	5.5	213.0849, 185.0811	Lactucin	C15H16O5	276.2845*	Lactone
5	4.66	471.1096	471.1073	4.9	454.0898, 427.1523, 391.2080, 356.0896, 339.0364, 131.0306	Lactucin-sulfate	C19H24N2O10S	472.1079	Lactone derivative
6	6.46	339.0517	339.0538	-6.2	96.9466	15-deoxylactucin-8-sulfate	$C_{15}H_{16}O7S$	340.0544	Lactone
7	4.79	485.1276	485.123	9.5	405.1581, 427.1523, 391.2080, 356.0974, 339.0592, 145.0742	15-deoxylactucin-8-sulfate-Gln	C20H26N2O10S	486.1235	Lactone derivative
8	4.94	454.1203	454.1172	6.8	339.0554, 114.0487	15-deoxylactucin-8-sulfate-Pro	C20H25NO9S	454.1177	Lactone derivative
9	4.07	456.1378	456.1328	11.0	356.0703, 339.0479, 116.0885	15-deoxylactucin-8-sulfate-Val	C20H27NO9S	457.1334	Lactone derivative
10	6.57	341.0674	341.0695	-6.2	96.9415	8-deacetylmatricarin-8-sulfate	$C_{15}H_{18}O7S$	342.07	Lactone
11	4.4	519.1203	519.1172	6.0	501.0999, 475.1301	Cichorioside B-sulfate	C21H28O13S	520.1178	Lactone derivative
12	1.73	561.2349	561.2336	2.3	427.1185, 409.1131, 151.0492	1β-(4-hydroxyphenylacetyl) -15-O-β-D-glucopyranosyl-5α, 6βH-eudesma-3-en-12, 6α-olide	C29H38O11	562.2341	Lactone

*Compared by database entries.

Table S3 LC-MS characteristics of non-phenolic metabolites putatively identified in *Lactuca sativa*

No.	RT (min)	Observed m/z ([M-H]⁻)	Calculated m/z ([M-H]⁻)	Error (ppm)	Major Fragments m/z ([M-H]⁻)	Putative compound	Empirical formula	Empirical weight	Class
1	1.65	130.0834	130.0868	-26.1	112.0724, 86.0967	Leucine/Isoleucine	$C_6H_{13}NO_2$	131.1729*	Amino Acid
2	4.29	259.1307	259.1294	5.0	215.1426, 171.1512, 130.0881	Glycine-Leucine	C11H19N2O5	259.279*	Amino Acid
3	4.82	421.1828	421.1822	1.4	331.1492, 241.1147, 218.0829, 130.0834, 128.0347	Glycine-Leucine/Isoleucine-hexose	C17H29N2010	422.1828	Amino Acid
4	3.93	203.0817	203.0821	-2.0	159.0829, 142.0648, 116.0466, 74.0298	Tryptophan	C11H12N2O2	204.2252*	Amino Acid
5	7.54	365.1309	365.1349	-11.0	203.0876, 159.0854	Tryptophan-hexose	C17H22N207	366.1354	Amino Acid
6	1.14	180.064	180.0661	-11.7	163.0366, 119.0537, 93.0424	Tyrosine	$C_9H_{11}NO_3$	181.1885*	Amino Acid
7	5.26	342.1191	342.1189	0.6	180.0668	Tyrosine-hexose	C15H21NO8	343.1194	Amino Acid
8	3.11	164.068	164.0712	-19.5	147.0433, 103.0478, 72.0113	Phenylalanine	C9H11NO2	165.1891*	Amino Acid

9	2.85	326.1211	326.124	-8.9	236.0969, 206.0798, 164.0706, 147.0409	Phenylalanine-hexose	C15H21NO7	237.1245	Amino Acid
10	5.7	133.011	133.0137	-20.3	115.0016, 71.0125	Malic acid	C4H6O5	134.0874*	Fatty Acyls
11	1.1	147.026	147.0293	-22.4	103.027	Citramalic acid	C5H8O5	148.114*	Fatty Acyls
12	0.89	128.0324	128.0348	-18.7	82.0264	Pyroglutamic acid	C5H7NO3	129.114*	Carboxylic acids
13	5.73	241.1179	241.1188	-3.7	197.1286, 130.0834	Pyroglutamic acid-Leucine/Isoleucine	C11H18N204	242.1194	Amino Acid
14	4.63	175.059	175.0606	-9.1	157.0498, 115.0477, 113.0540, 85.0591	Isopropylmalic acid	C7H12O5	176.1672*	Fatty Acyls
15	8.11	187.0944	187.097	-13.9	169.0822, 143.1036, 125.0918	Azelaic acid	C9H16O4	188.2209*	Fatty Acyls
16	5.57	191.0545	191.0556	-5.8	87.0471	Quinic acid	C7H12O6	192.1666*	Alcohols & polyols
17	0.78	191.0177	191.0192	-7.9	111.0088, 87.0032	Citric Acid	C6H8O7	192.1235*	Carboxylic acids
18	7.15	186.1101	186.113	-15.6	142.1234, 125.0963	Amino oxononanoic acid	C9H17N03	187.1136	Organic acid
19	2.33	218.1011	218.1028	-7.8	148.0794, 88.0450, 71.0505	Pantothenic acid (vitamin B5)	C9H17NO5	219.235*	Carboxylic acids
20	1.09	243.0607	243.0617	-4.1	200.0535, 152.0319, 140.0325, 110.0211	Uridine	$C_9H_{12}N_2O_6$	244.2014*	Pyrimidine nucleosides

21	0.73	266.0865	266.0889	-9.0	134.0470, 107.0251	Adenosine	C10H13N5O4	267.2413*	Purine nucleosides
22	1.9	282.0835	282.0838	-1.1	150.0395, 133.0110, 108.0248	Guanosine	C10H13N5O5	283.2407*	Purine nucleosides
23	4.82	215.0826	215.0821	2.3	171.0897, 142.0648, 116.0488	1,2,3,4-Tetrahydro-beta-carboline-3-carboxylic acid	C12H12N2O2	216.2359*	Indoles and derivatives
24	4.83	259.0714	259.0719	-1.9	215.0826, 171.0897, 142.0648	1,2,3,4-Tetrahydro-b-carboline-1,3-dicarboxylic acid	C13H12N2O4	260.2454*	Harmala alkaloids

*Compared by database entries.

Table S4 LC-MS characteristics of phenolic metabolites putatively identified in *Lactuca sativa*

No.	RT (min)	Observed m/z ($[M-H]^-$)	Calculated m/z ($[M-H]^-$)	Error (ppm)	Major Fragments m/z ($[M-H]^-$)	Putative compound	Empirical formula	Empirical weight	Class
1	3.74	137.0234	137.0239	-3.6	120.9012, 92.9595	Hydroxybenzoic acid	C7H6O3	138.1207*	Hydroxybenzoic acid
2	2.77	153.0178	153.0188	-6.5	153.0178, 109.0164	Dihydroxybenzoic acid	C7H6O4	154.1201*	Hydroxybenzoic acid
3	2.84	301.0923	301.0923		139.0266	3-Methoxy-4-hydroxyphenyl-1-O-β-D-glucoside	C13H18O8	302.0929	Hydroxybenzoic acid
4	2.96	197.0452	197.0450	1.0	182.0278, 153.0507, 138.0388	Syringic acid	C9H10O5	198.1727*	Hydroxybenzoic acid
5	5.82	167.0336	167.0344	-4.8	151.9360, 107.9908	Vanillic Acid	C8H8O4	168.1467*	Hydroxybenzoic acid
6	1.96	329.0851	329.0873	-6.7	167.0389, 23.0443, 108.0248	Vanillic acid glucoside	C14H18O9	330.0878	Hydroxybenzoic acid
7	4.21	359.0973	359.0978	-1.4	197.0452, 182.0222, 153.0583, 138.0292, 123.0103	Syringic acid hexose	C15H20O10	360.0984	Hydroxybenzoic acid
8	3.15	315.0712	315.0716	-1.3	153.0092, 108.0248	Dihydroxybenzoic acid hexose	C13H16O9	316.0722	Hydroxybenzoic acid
9	7.28	435.0916	435.0927	-2.5	315.0821, 297.0704, 153.0204, 152.0168, 137.0186	Hydroxybenzoyl dihydroxybenzoyl-hexose	C20H20O11	436.0933	Hydroxybenzoic acid
10	4.87	179.0354	179.0344	5.6	135.0442	Caffeic acid	C9H8O4	180.1574*	Hydroxycinnamic acid
11	3.4	343.1004	343.1029	-7.3	181.0459, 163.0392, 135.0418	Dihydrocaffeic acid hexose	C15H20O9	344.1035	Hydroxybenzoic acid

12	4.56	341.0863	341.0859	1.2	n.d	Caffeoyl-hexose	C15H18O9	342.0878	Hydroxycinnamic acid
13	7.45	193.0487	193.0501	-7.3	178.0262, 149.0583, 134.0328	Ferulic acid	C10H10O4	194.184*	Hydroxycinnamic acid
14	8.73	207.0624	207.0657	-15.9	192.0149, 177.0171	Ferulic acid methyl ester	C11H12O4	208.0663	Hydroxycinnamic acid
15	5.97	355.1015	355.1029	-3.9	193.0686, 149.0633	Ferulic acid glucoside	C16H20O9	356.1035	Hydroxycinnamic acid
16	5.24	385.1144	385.1135	2.3	223.0594, 208.0355, 179.0354, 164.0444	Sinapoyl glucoside	C17H22O10	386.1140	Hydroxycinnamic acid
17	4.99	325.0924	325.0943	-5.8	163.0392, 119.0493	p-Coumaroyl glucoside	C15H18O9	326.0929	Hydroxycinnamic acid
18	3.44	311.0366	311.0403	-11.9	179.0327, 145.0058, 135.0418	Caftaric acid	C13H12O9	312.229*	Hydroxycinnamic acid
19	3.45	149.0058	149.0086	-18.8	103.0041, 81.0032, 72.9943, 59.0966	Tartaric acid	C4H6O6	150.0868*	Carbohydrates
20	5.01	353.087	353.0873	-0.8	191.0545, 179.0327, 135.0418	Caffeoylquinic acid	C16H18O9	354.3087*	Hydroxycinnamic acid
21	5.71	295.0457	295.0454	1.0	163.0366, 119.0537	Coutaric acid	C13H12O8	296.2296*	Hydroxycinnamic acid
22	6.29	337.0908	337.0923	-4.4	191.0545, 173.0444, 163.0392	p-Coumaroylquinic acid	C16H18O8	338.3093*	Alcohols and polyols
23	5.5	515.1172	515.119	-3.5	353.0870, 191.0545, 179.0327, 135.0442	Dicaffeoylquinic acid	C25H24O12	516.4509*	Alcohols and polyols

24	6.71	457.0783	457.0771	2.6	311.0402, 295.0422, 277.0388, 179.0327, 163.0366, 149.0208	Caffeoyltartaric-p-coumaroyl acid	C22H18O11	458.0776	Hydroxycinnamic acid
25	7.83	499.1224	499.124	-3.2	353.0870, 191.0545	p-Coumaroyl-caffeoylquinic acid	C25H24O11	500.1246	Hydroxycinnamic acid
26	4.56	313.0905	313.0923	-5.7	151.0391, 107.0391	4-hydroxyphenylacetyl glucoside	C14H18O8	314.0929	Hydroxyphenylacetic acid
27	0.53	447.1289	447.1291	-0.4	193.0317, 175.0238, 151.0466	Di(4-hydroxyphenylacetyl)-hexose	C22H24O10	448.1297	Hydroxyphenylacetic acid
28	8.86	301.0319	301.0348	-9.6	178.9752, 151.0039	Quercetin	C15H10O7	302.2357*	Flavonoids
29	6.55	463.0846	463.0877	-6.7	301.0319, 300.0241	Quercetin hexose	C21H20O12	464.0882	Flavonoids
30	7.27	477.064	477.0669	-6.1	301.0319	Quercetin 3-glucuronide	C21H18O13	478.0675	Flavonoids
31	7.63	549.086	549.088	-3.6	505.0989, 463.0846, 301.0319	Quercetin malonylglucoside	C24H22O15	550.0886	Flavonoids
32	5.84	531.1142	531.1139	0.6	463.1022, 301.0426, 300.0348	Quercetin-3-O-(6"-O-crotonyl)-β-glucoside	C25H23C13	531.1144	Flavonoids
33	7.05	579.1681	579.1714	-5.7	271.0613	Naringenin 7-neohesperidoside	$C_{27}H_{32}O_{14}$	580.1792*	Flavonoids
34	7.81	445.0753	445.0771	-4.0	269.0464	Apigenin 7-O-glucuronide	$C_{21}H_{18}O_{11}$	446.0849*	Flavonoids
35	7.66	665.2063	665.2082	-2.9	417.1554	Syringaresinol malonylhexose	C31H38O16	665.2087	Lignan
36	5.77	579.2075	579.2078	-0.5	417.1847, 399.1428, 384.1068	Syringaresinol-β-D-glucoside	C28H36O13	580.2083	Lignan

37	9.77	581.2192	581.2234	-7.2	359.1671, 341.1921, 329.1407	Alangilignoside C	C28H38O13	582.2240	Lignan
38	7.35	341.1241	341.1236	1.5	179.0683	Coniferoside	C16H22O8	342.3411*	Monolignol
39	2.54	405.1152	405.1186	-8.4	243.0703	2,3,5,4β-Tetrahydroxystilbene2-O-β-D-glucoside	C20H22O9	406.1191	Stilbene
40	6.83	411.1278	411.1291	-3.2	163.0758, 148.0404	Eugenol malonylglucoside	C19H24O10	412.1297	Phenylpropene

*Compared by database entries.

© 2019 by the authors. Submitted for possible open access publication under the terms and conditions of the Creative Commons Attribution (CC BY) license (http://creativecommons.org/licenses/by/4.0/).
